# Supplementary material for: Metabolome and Mycobiome of Aegilops tauschii Subspecies Differing in Susceptibility to Brown Rust and Powdery Mildew Are Diverse
Source: Plants (Basel). 2024 Aug 23;13(17):2343. doi: 10.3390/plants13172343 (PMC11397189; doi:10.3390/plants13172343)
Supplement: Supplementary file 1 [file plants-13-02343-s001.zip › plants-3112123-supplementary.pdf]

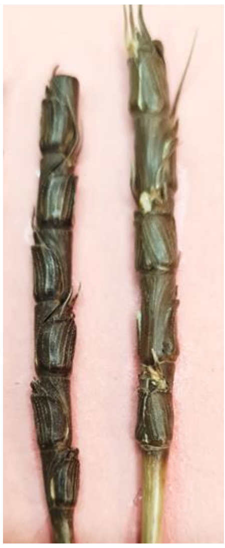

FigureS1. *Ae.tauschii* Coss.ssp.*meyeri* k-340.

Note: Collection of Federal Research Center All-Russian Institute of Plant Genetic Resources (VIR)

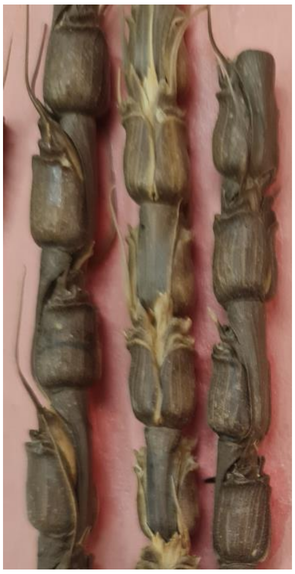

FigureS2. *Ae.tauschii* Coss.ssp.*strangulata* к-1958

Note: Collection of Federal Research Center All-Russian Institute of Plant Genetic Resources (VIR)

Table S1. Metabolomic analysis : the content of biological active substances in seeds of *Aegilops tauschii* 1958 accession (resistant) and 340 accession (sensible) to *Puccinia recondite and Blumeria graminis*

Substance	Abbreviation	340	1958
		Mean ± SE, ppm	Mean ± SE, ppm
**Organic acids**			
Lactic acid	lactic	5.81±0.16	6.28±0.01
Pyruvic acid	PYR	0.39±0.01	0.55±0.04
Methylmalonic acid	methylmalonic	11.83±0.21	12.99±0.38
Oxalic acid	oxalic	2.02±0.49	2.45±0.01
3Hydroxypropionic acid	3hydroxypr	0.19±0.04	0.03±0.004
Methylphosphonic acid	methylphosph	4.41±0.77	9.07±0.72
Benzoic acid	benzoic	0.02±0.002	0.01±0.004
Posphoric acid	posph	12.57±2.29	38.30±5.83
Nicotinic acid	nicotinic	0.11±0.001	0.17±0.07
Maleic acid	maleic	2.32±0.84	2.53±0.16
Succinic acid	succinic	2.54±0.02	1.68±0.16
Glyceric acid	glyceric	1.23±0.34	6.94±0.77
Fumaric acid	fumaric	2.01±0.37	1.94±0.11
Malic acid	malic	10.78±0.78	28.24±2.21
Salicylic acid	salicylic	0.13±0.05	0.36±0.06
Erythritic acid	erythritic	0.41±0.09	0.82±0.09
Pyrogallol	pyrogallol	0.12±0.001	0.40±0.05
2,3 Hydroxybenzoic acid	2,3 hydroxybenzoic	0.29±0.07	0.33±0.02
Azelaic acid	azelaic	1.86±0.03	2.72±0.28
Ribonic acid	ribonic	2.62±0.21	24.46±3.51
Galacturonic acid	GalUA	6.04±0.39	12.43±0.51
Caffeic acid	caffeic	0.21±0.04	0.60±0.01
Gluconic acid	gluconic	3.76±1.35	11.08±1.15
**Amino acids**			
Alanine	Ala	8.97±0.89	6.01±0.16
Glycine	Gly	0.96±0.08	1.38±0.09
Valine	Val	14.75±1.40	9.39±1.30
Isoleucine	Ile	1.08±0.20	1.33±0.001
Proline	Pro	2.23±0.39	2.31±0.02
Serine	Ser	1.40±0.95	1.50±0.001
Threonine	Thr	1.44±0.08	1.96±0.10
Oxyproline	OxPro	1.67±0.36	2.97±0.61
Aspartic acid	Asp	3.10±1.03	1.73±0.23
Glutamic acid	Glu	3.97±0.67	2.80±0.38
Glutamine	Gln	13.61±4.39	37.09±1.18
Phenilalanine	Phe	0.96±0.76	0.34±0.06
Asparagine	Asn	14.05±0.77	4.11±0.23
Tyrosine	Tyr	1.20±0.76	4.35±0.14
Tryptophan	Trp	0.68±0.31	0.82±0.03
**Carbonic acids**			
3 hydroxypipecolic	3 hydroxypipecolic	133.59±23.03	135.61±4.60
pipecolic	pipecolic	0.34±0.01	0.51±0.04
5hydroxypipecolic	5hydroxypipecolic	0.69±0.20	0.61±0.11
**Polyhydric alcohols**			
Glycerol	glycrl	59.68±6.37	76.71±5.14
Threitol	threitl	2.89±0.56	1.40±0.22
Arabinitol	arabl	60.94±7.72	48.34±6.36
Dulcitole	dulcl	30.90±6.64	55.03±2.72
Mannitol	manntl	22.58±3.61	26.73±0.24
Chiro-inositol	chr-inostl	5.00±1.04	4.89±0.34

Continuation **Table S1**.

Myoinositol	mInosl	15.98±2.67	67.33±4.76
Galactinol	galtl	77.58±4.48	151.05±14.11
**Phytosterols**			
Stigmasterol	stigstrl	5.23±2.07	4.27±0.40
Campesterol	campstrl	1.41±0.5	2.35±0.12
Sitosterol	sitostrl	186.34±41.95	304.23±16.43
**Fatty acids**			
Pelargonic acid	pelargonic	2.37±1.10	0.77±0.12
Undecylic acid	undecylic	10.99±5.55	12.56±1.88
Palmitic acid	palmitic	67.31±8.43	83.15±2.94
Hydroxyoctodecanoic acid	hydroxyoctodecanoic	0.33±0.009	0.57±0.09
Linolenic acid	linolenic	169.54±1.48	188.79±7.58
Oleic acid	oleic	46.31±0.37	60.91±1.08
Vaccenic acid	vac	6.36±1.07	7.64±0.99
Stearic acid	stearic	22.55±2.29	21.05±2.04
Eicosanoic	eicosanoic	1.77±0.21	0.71±0.20
Behenic acid	behenic	3.05±0.48	3.33±0.30
Hydroxyoctacosenic acid	hydroxyoctacosenic	0.18±0.001	0.45±0.03
Hydroxyhexacosenicacid	hydroxyhexacosenic	0.36±0.11	0.27±0.04
**Glycerols**			
Monoacylglycerol 16:0	MAG1	8.43±2.40	6.58±0.13
Monoacylglycerol 18:2	MAG2	22.31±0.74	14.31±0.53
Monoacylglycerol 18:0	MAG3	4.00±0.98	2.83±0.04
Diacylglycerol	DAG	45.77±1.16	55.61±1.78
**Sugars**			
Glyceraldehyde	glAld	17.36±1.67	29.42±2.86
Ribose	Rib	4.34±1.07	3.16±0.08
Xylose	Xyl	0.29±0.11	0.40±0.05
Fructose	Fruct	46.05±14.61	128.02±6.94
Sorbose	Sorb	17.19±5.09	6.62±1.95
Galactose	Gal	3.37±0.44	39.10±2.82
Glucose	Gluc	226.00±4.16	6888.81±323.00
Mannose	Mann	1.82±0.18	3.49±0.35
Sucrose	Suc	12219.44±1154.47	16378.00±1050.96
Maltose	Malt	8.00±0.06	13.34±0.33
Melibiose	Melib	2.04±0.86	0.63±0.11
Raffinose	Raff	5302.27±449.68	2000.00±230.28
Stachyose	Stach	1.48±0.21	3.66±0.06
**Glycosides**			
Glycoside1 Galactopyranoside	glysd 1	0.12±0.003	0.61±0.18
Glycerol –d- Galactopyranoside	glysd 2	10.04±0.49	19.96±0.40
**Nucleoside**			
Adenosine	Adenosine	0.26±0.05	2.86±0.24
**Secondary metabolites**			
Hydroqhenone	hydrqn	87.04±2.75	78.09±3.29
a-tocopherol	a-tocophls	0.08±0.01	0.18±0.021
Kaempeferol	kaempfrl	18.52±1.41	16.25±1.29
Salicylic acid*	salicylic	0.13±0.008	0.36±0.06
Pyrogallol*	pyrogallol	0.12±0.001	0.40±0.05
2,3 Hydroxybenzoic acid*	2,3 hydroxybenzoic	0.29±0.07	0.33±0.02
Caffeic acid*	caffeic	0.21±0.04	0.60±0.10

Notes: Secondary metabolites marked with an asterisk have already been classified above

Table S2. Features and pathogenicity of identified fungi of *Aegilops tauschii* seeds

Identified genera and species of fungi	Relative abundance, %		Growth morfology	Suggested genus function	Wheat disease	References
	k-1958	k-340				
Alternaria	19.06±6.56	39.22±2.35	Filamentous fungi	Saprotroph, Pathogen	black point	
Alternaria infectoria (E.G. Simmons)	15.53±5.19	30.67±2.43	Filamentous fungi	Pathogen	black point	~~[85-87]~~ [84-86]
Blumeria	3.98±2.91	38.58±1.90	Filamentous fungi	Pathogen		
Blumeria graminis (de Candolle) Speer.	0	7.30±1.99	Filamentous fungi	Pathogen	powdery mildew	~~[88-90]~~ [87-89]
Cladosporium	47.14±22.11	12.35±3.07	Filamentous fungi	Saprotroph, Pathogen	black point	[92-94] [91-93]
Vishniacozyma	6.15±3.35	3.58±0.49	Yeast	Saprotroph		
Vishniacozyma victoriae (M.J. Montes, Belloch, Galiana, M.D. Garca, C. Andrs, S. Ferrer, and Torr.-Rodr. and J. Guinea)	3.44±2.07	0.90±0.16	Yeast	Biocontrol agent		~~[111]~~ [108]
Vishniacozyma tephrensis (Vishniac) Xin Zhan Liu, M. Groenew& Boekhout	0.54±0.28	0.58±0.06	Yeast	Biocontrol agent		~~[111]~~ [108]
Vishniacozyma dimennae (Fell & Phaff) Xin Zhan Liu, F.Y. Bai, M. Groenew & Boekhout	0.51±0.35	0.03±0.03	Yeast			
Vishniacozyma foliicola (A.M. Yurkov)	0.01±0.01	0	Yeast			
Sporobolomyces	2.06±1.55	0.40±0.04	Yeast	Saprotroph		~~[112]~~ [109]
Sporobolomyces roseus (Kluyver& C.B. Neil)	2.06±1.55	0.40±0.04	Yeast	Biocontrol agent		~~[113]~~ [110]
Stemphylium	1.30±0.50	1.01±0.10	Filamentous fungi	Pathogen		~~[95]~~ [94]
Parastogonospora	0	1.28±0.83	Filamentous fungi	Pathogen		~~[96]~~ [95]
Parastagonospora avenae (F.B. Frank) (Quaedvlieg, Verkley & Crous)	0	0.82±0.41	Filamentous fungi	Pathogen	yellow leaf spot	~~[98,99]~~ [97,98]
Parastagonospora phragmitis (Crous & Y. Marín)	0	0.40±0.40	Filamentous fungi	Pathogen Wild grasses	n/d	~~[100]~~ [99]
Puccinia	0	0.14±0.07	Filamentous fungi	Pathogen		~~[101]~~ [24]

Continuation **Table S2.**

Puccinia recondita (Rob. ex Desm. f. sp. tritici)	0	0.11±0.07	Filamentous fungi	Pathogen	brown rust	~~[102]~~ [100]
Puccinia striiformis (Westendorp.)	0	0.03±0.01	Filamentous fungi	Pathogen	stripe yellow rust	~~[103]~~ [101]
Acremonium	0.15±0.08	0.75±0.32	Filamentous fungi	Saprotroph		~~[111]~~ [108]
Acremonium alternatum	0.15±0.08	0.46±0.30	Filamentous fungi	Hyperparasite Biocontrol agent		~~[114,115]~~ [111-112]
Gibberella	0.21±0.21	0.01±0.01	Filamentous fungi	Pathogen		~~[107]~~ [105]
Giberella intricans (Wollenw)	0.21±0.21	0.01±0.01	Filamentous fungi	Pathogen		~~[107]~~ [105]
Selenophoma	0	0.18±0.18	Filamentous fungi			
Selenophoma linicola (Vanterpool)	0	0.18±0.18	Filamentous fungi	Flax Pathogen,	n/d	~~[110]~~ [107]
Fusarium	0.10±0.06	0	Filamentous fungi	Pathogen		
Fusarium langsethiae (Torp & Nirenberg)	0.10±0.06	0	Filamentous fungi		head blight	~~[108]~~ [106]
Dioszegia	0.14±0.07	0.05±0.01	Filamentous fungi			
Dioszegia hungarica (Zsolt.)	0.14±0.07	0.05±0.01	Yeast	Pathogen		~~[109]~~ [95]
Cystofilobasidium	0.03±0.02	0.12±0.12	Yeast	Saprotroph		
Cystofilobasidium macerans (Samp)	0.03±0.02	0.12±0.12	Yeast			
Beauveria	0.37±0.37	0		Entomopathogen		
Beauveria bassiana (Balsamo)	0.37±0.37	0		Entomopathogen Biocontrol agent		~~[116]~~ [113]

TableS3. Passport data of *Ae.tauschii* accessions from Collection of Federal Research Center N. I. Vavilov All-Russian Institute of Plant Genetic Resources (VIR)

Catalog Number	Country	Region	Expedition, year	Family	H/altitude*
K-340	Azerbaijan	Nagorno-Karabakh, village Garov	1961, VIR	Dorofeev V.F.	1300
K-1958	Iran	Gilan, town Resht	1976 Institute of Genetics and Selection of Azerbaijan	Mustafaeva I.D.	1350

Note: *height above sea level

Table S4. Characteristics of economically useful traits of the original accessions of *Ae.tauschii* from the VIR collection

**Specimen Catalog Number**	**Plant height, cm**	**Heading date**	**Resistance score to *Puccinia recondita***	**Resistance score to** *Blumeria graminis*
k-340	70-75	12.05- 20.05	**3**	**1-3**
k-1958	70-75	12.05- 20.05	**9**	**9**

Note: Data based on the results of long-term field data (1991– 2023)

Table S5. Scale of the degree of plant damage by brown rust (*Puccinia recondita*)

Score	Degree of resistance	Presence of pustules	Affected leaf surface, %
9	Very high	Lesions are absent or single, very small, surrounded by necrosis	<5
7	High	Small, sometimes surrounded by chlorosis, scattered on leaves and stems	<10
5	Average	Small, with or without chlorosis	-20
3	Low	Numerous, merging, especially abundant on the middle tier of leaves	-50
1	Very low	Densely spaced, large, confluent, mostly on the middle and often on the upper tier of leaves and stems	>50
